# The Psychometric Properties of the French Version of the Personality Inventory for DSM-5

**DOI:** 10.1371/journal.pone.0133413

**Published:** 2015-07-20

**Authors:** Isabelle Roskam, Sarah Galdiolo, Michel Hansenne, Koorosh Massoudi, Jérôme Rossier, Ludovic Gicquel, Jean-Pierre Rolland

**Affiliations:** 1 Psychological Sciences Research Institute, University of Louvain, Louvain-la-Neuve, Belgium; 2 Department of Psychology, University of Liège, Liège, Belgium; 3 Institute of Psychology, University of Lausanne, Lausanne, Switzerland; 4 Unité de Recherche Clinique, Centre Hospitalier Spécialisé Henri Laborit, Saint-Benoît, France; 5 Université Paris Ouest La Défense, Nanterre, France; Georgia State University, UNITED STATES

## Abstract

In the context of the publication of DSM-5, the Personality Inventory for DSM-5 (PID-5) has been proposed as a new dimensional assessment tool for personality disorders. This instrument includes a pool of 220 items organized around 25 facets included in a five-factor second-order domain structure. The examination of the replicability of the trait structure across methods and populations is of primary importance. In view of this need, the main objective of the current study was to validate the French version of the PID-5 among French-speaking adults from a European community sample (N=2,532). In particular, the assumption of unidimensionality of the 25 facet and the five domain scales was tested, as well as the extent to which the five-factor structure of the PID-5 and the DSM-5 personality trait hierarchical structure are replicated in the current sample. The results support the assumption of unidimensionality of both the facets and the domains. Exploratory factor and hierarchical analyses replicated the five-factor structure as initially proposed in the PID-5.

## Introduction

In the context of the publication of DSM-5, a new dimensional assessment tool for personality disorders has been proposed, the Personality Inventory for DSM-5 (PID-5). Its purpose is to be widely available in the public domain as a self-report and informant-report personality inventory [1]. The experts involved in the DSM-5 Personality and Personality Disorders Work Group explored previous models and corresponding assessment instruments focused on pathological personality, such as the Dimensional Assessment of Personality Pathology [2], the Personality Psychopathology Five (PSY-5) model [3], the Dimensional Personality Symptom Item Pool (DIPSI) Model [4], and structural models of DSM Disorders [5]. This approach led them to start with 37 facet descriptions and to derive an empirical structure of personality pathology encompassing several broad domains, i.e. neuroticism, detachment, disinhibition, antagonism, compulsivity and psychoticism [1]. Preliminary data collection from a thousand subjects resulted in a pool of 220 items organized around 25 facets included in a five-factor second-order domain structure, i.e. Negative Affectivity, Detachment, Antagonism, Disinhibition and Psychoticism [6]. Conceptually, definitions of facets and domains were provided as well as the expected hierarchical model of variation in personality and psychopathology [7].

There was then a need for additional research in order to add detail and substance to DSM-5 and subsequent revisions [6]. The importance of examining the replicability of the trait structure across methods and populations has been stressed by many authors [8]. In view of this need, the main objective of the current study was to report data about the factorial structure of the French version of the PID-5. In particular, it was an attempt to test the assumption of unidimensionality of the 25 facet and the five domain scales, as well as the extent to which the five-factor structure of the PID-5 and the DSM-5 personality traits hierarchical structure are replicated among French-speaking adults from a European community sample.

In order to inform the hypotheses of unidimensionality of facets and domains, five-factor structure, and personality traits hierarchical structure, we selected published research where at least one of these had been tested. The current literature review was limited to these relevant studies: those concerning convergent validity or joint hierarchical analyses, for example, were not used. A synthesis of the selected studies is presented in Table 1.

**Table 1:** Synthesis of the literature review.

Authors	Date	Version	Sample	Facet and domain unidimensionality	Five-factor structure replication	Hierarchical structure
Krueger et al.	2012	English SRF	N = 264 adults	Cronbach’s alphas	Exploratory Factor Analysis (EFA)	
			CS			
Bastiaens et al.	2015	Dutch SRF	N = 240 adults	Confirmatory Factorial Analyses (CFAs)	Exploratory Structural Equation Modeling (ESEM)	
			CR		Congruence coefficients	
Bo et al.	2015	Danish SRF	N = 1119 adult patients	Cronbach’s alphas	Parallel analysis	Goldberg (2006)
			CS and CR	Item-total-correlation	EFA	
					Congruence coefficients	
De Clerck et al.	2014	Dutch SRF	N = 434 adolescents	Cronbach’s alphas	Parallel analysis	
			CS		Minimum Average Partial tests (MAP)	
					EFA	
					Congruence coefficients	
					Exploratory Structural Equation Modeling (ESEM)	
De Fruyt et al.	2013	Dutch SRF	N = 444 students	Cronbach’s alphas	EFA	
			CS		Congruence coefficients	
Fossati et al.	2013	Italian SRF	N = 710 adults	Cronbach’s alphas	CFA	
			CS	McDonald’s omega		
				Parallel analyses		
				CFAs		
Markon et al.	2013	English IRF	N1 = 320 adults	Parallel analysis	Parallel analysis	
			N2 = 40 adults	Cronbach’s alphas	MAP	
			N3 = 221 at risk adults	McDonald’s omega	ESEM	
			CS			
Morey et al.	2013	English CRF	N = 337 adult patients	-	-	Goldberg (2006)
			CR			
Quilty et al.	2013	English SRF	N = 201 adult patients	Cronbach’s alphas		
			CR	McDonald’s omega		
				Average Item Correlation (AIC)		
				MAP		
Wright et al.	2012	English SRF	N = 2,461 students		EFA	Goldberg (2006)
			CS			
Zimmerman et al.	2014	German SRF	N = 577 students	Cronbach’s alphas	EFA	
			CS			
			N = 212 adult patients	CFAs	Congruence coefficients	
			CR			

Note: CS community sample; CR clinically referred; SRF Self-Report Form; CRF Clinician Report Form; IRF Informant Report Form

In these studies, the unidimensionality of the 25 facet scales was appraised using several methods: reliability analyses, parallel analyses or Velicer’s minimum average partial (MAP) tests, and confirmatory factor analyses (CFAs). In all studies under consideration, the assumption of unidimensionality was mainly supported by the findings. However the unidimensionality was questionable for Suspiciousness in the study of De Clercq et al. conducted with a Dutch self-report form of the PID-5 [9], Risk Taking in both the English and the Italian self-report forms as well as in the English informant-report form [10–12], Hostility in both the German and the English forms [10, 13], Emotional Lability in both the German and the Dutch forms [13, 14], Perseveration and Manipulativeness in the German self-report form [13], and Depressivity in the English informant-report form [10, 12]. Overall, relatively strong support for the hypothesis of unidimensionality of the scales emerges from these results and leads us to expect similar findings.

With regard to the five-factor structure replication, most of the authors reproduced the analysis strategy used in the initial validation study, i.e. equamax rotated exploratory factor analysis (EFA). In several cases, EFA was preceded by parallel analyses or MAP tests in order to find out how many factors to retain. A five-factor structure emerged from the parallel analyses conducted on the Italian self-report form [11]. On several occasions, it was suggested that two, three, four or six factors should be retained [9, 12, 15, 16], but in these cases the five-factor solution was preferred on the basis of goodness-of-fit indices [9], or presented to enable a direct comparison with the initial structure [15] or as the most clearly interpretable solution [12, 16]. EFAs mainly replicated the primary and cross-loadings found by Krueger et al. (2012) [16, 17]. In Zimmerman et al. (2014), Bastiaens et al. (2015) as well as in Wright et al. (2012), however, the primary loading of Hostility was on Antagonism rather than on Negative Affectivity as expected. In addition, in Zimmerman et al. (2014) and Wright et al. (2012), the primary loading of Restricted Affectivity was on Detachment rather than on Negative Affectivity as expected. The same was observed in the Danish self-report version [15]. In this version, Rigid Perfectionism also loaded predominantly on the Negative Affectivity factor [15]. Also, Disinhibition did not replicate well in the adult patients sample [13]. In addition to the EFAs, Tucker’s congruence coefficients enabled several authors to compare the initial factor structure displayed by Krueger et al. (2012) with their findings. Good to high similarity was found, especially between the English and the Dutch, Danish and German versions [9, 13, 15, 17]. According to these previous results, the five-factor structure should be replicated with the French version of the PID-5. Similarity is also expected with the initial structure of the instrument [6].

The multidimensional reconceptualization of personality pathology undertaken for the PID-5 [1, 5, 18] recognizes a certain overlap between pathological and normal personality variations as they are recorded in the Five-Factor Model of Personality [19, 20] and this overlap holds across cultures [21]. As suggested by Widiger and Simonsen, both normal personality functioning and pathological personality could be integrated in a hierarchical model with two higher-order domains of Internalizing and Externalizing behavior corresponding to the model of general psychopathology [22–24]. In this respect, several authors have explored the hierarchical structure of the traits using the method suggested by Goldberg [25]. This is based on the estimation of a series of factor models with an increasing number of factors. The across-model correlations are then used to estimate the paths between levels of the hierarchy [24]. To the best of our knowledge, only three studies referred to this method in order to replicate the initial five factors [8, 15, 16]. At the two-factor level, Internalizing and Externalizing were found in these three studies. At the three-factor level, Externalizing behavior replicated whereas Internalizing behavior split into Detachment and Negative Affect. This level has been thought to correspond to the Big Three of the literature on temperament [26, 27]. The fourth level was characterized by the split of Externalizing behavior into Disinhibition and Antagonism [15, 16]. It was considered to be close to the Dimensional Assessment of Personality Pathology (DAPP-BQ) [28, 29]. The results found by Morey et al. (2013), however, diverged from those of Bo et al. (2015) and Wright et al. (2012) at the fourth level. The split of Externalizing factor into two factors did not occur. Instead, Detachment split into Detachment and Psychoticism. Whereas the five domains were found and replicated in both the English and Danish self-report forms [15, 16], Antagonism and Disinhibition could not be replicated with the English clinician-report form [8]. In the current study, the hierarchical structure of DSM-5 traits will be explored. Internalization and Externalization are expected at the top of the hierarchy with the pathological five-factor model at lower levels.

In the current study, the French version of the self-report form of the Personality Inventory for DSM-5 (PID-5) was administered to French-speaking adults from a European community sample encompassing subsamples from Belgium, France and Switzerland. In the data analysis, an attempt was first made to test the assumption of unidimensionality of both the facet and the domain scales. We then tried to replicate the initial five-domain structure of the PID-5 [6]. Finally, Goldberg’s approach was used to replicate the hierarchical structure of the traits, delineating a model with higher-order dimensions of Internalizing and Externalizing under which three to five broad dimensions correspond to fundamental dimensions of personality functioning [7, 16]. Given the findings from previous cross-validation studies, we expected to support the unidimensionality hypothesis as well as to replicate the initial five-factor structure.

## Method

### Sample

At the outset of the study, 2,648 adults were willing to participate. Of these, 2,532 (25.9% men) provided complete data (95.62%): 1,593 came from the French-speaking part of Belgium (62.9%), 536 were French-speaking Swiss (21.2%), and 403 were French (15.9%). Their ages ranged from 18 to 85 years (*M* = 27.22, *sd* = 13.28). Statistical analyses were computed with the complete data sets of these 2,532 participants.

For the French sample, the participants were psychology undergraduate volunteers from the University of Poitiers. The questionnaire was completed during group sessions at the university or individually by students who had volunteered to participate in the data collection. For the Belgian sample, exactly the same data collection procedure as in the French sample was used at the University of Liège among undergraduate volunteers. For the French-speaking part of Belgium, additional participants were also recruited at the University of Louvain by means of an announcement on websites, forums and social networks. The questionnaire was completed online, ensuring a dataset without missing data. For the Swiss sample, 289 participants were undergraduate volunteers from the University of Lausanne, while 247 others were recruited amongst the general population of students following a bachelor program at the University of Lausanne.

### Ethics Statement

Participation in the study was voluntary and participants could quit the study at any time they wished. Participants were aware that information would be used in a scientific study. Identifying information about the participants was kept under lock and key at a different place than the dataset itself by the principal investigator in each country. For a self-report research with adults from a community-sample, the approval by Ethics Committee was not required at the time the study was conducted for the three countries involved. Institutional review boards exempt researchers from approaching the committee for such kind of research.

### Instrument

The PID-5 is a 220-item self-report. Each item is assessed with a 4-point Likert-type scale ranging from 0 (very false or often false) to 3 (very true or often true). The French version of the PID-5 [30] has been developed using a translation/back-translation procedure. Several independent native French-speakers translated the original English items [6] into French. Two of them were French (J.D. Guelfi and J.-P. Rolland) and the remaining three were Belgian (G. Rossi, L. De Page, and E. Hennequin). Complete agreement (100% correspondence) between the five translators involved was required for the selection of the French items. Once agreement had been achieved for all 220 items, the initial French version of the inventory was back-translated from French into English by a translator who was unaware of the initial version of the PID-5. This first back-translation was then sent to two of the authors of the initial version, R.F. Krueger and K.E. Markon. Complete agreement was achieved.

### Analysis strategy

The assumption of unidimensionality of the 25 facet and the five domain scales was tested with both parallel analyses and reliability analyses computed with SPSS 22.0. Parallel analyses based on 1,000 random permutations of the original data were used. They make it possible to determine how many factors to extract for each facet and domain scale [31]. They are based on a comparison between eigenvalues from a factor analysis of the actual data and eigenvalues from a factor analysis of a random dataset. The number of factors to be retained is based on the number of actual data eigenvalues higher than the upper 95% confidence limit of random data eigenvalues [31]. To evaluate the internal consistency of the scales, we calculated Cronbach’s alpha coefficients (α). Descriptive statistics were then produced for the 25 facets and the five domains. A comparison with those reported in the initial validation study of the PID-5 [6] was made based on Cohen’s d.

Second, to examine if the PID-5 factor structure replicates in the current sample, we performed parallel analyses and subjected the 25 facet scales to an exploratory factor analysis (EFA) (equamax oblique rotation) with unweighted least squares as the method for factor extraction with Factor 9.3.1 [32]. This analysis provided goodness-of-fit indices in addition to the chi-square model, i.e. the comparative fit index (CFI), and the goodness-of-fit index (GFI) which should be higher than .90, and the root mean square residual (RMR) with perfect model fit being indicated by RMR = 0, and increasingly high values indicating worse fit [33, 34]. We also computed Tucker’s congruence coefficients [35] in order to compare Krueger’s five-factor solution [6] with the one found in the current sample. Usually, one of the solutions is subjected to a procrustean rotation, which makes it as similar as possible to the other solution. Since this procedure should make the coefficients of congruence even greater than they would be, it has not been employed in the current study. Tucker’s congruence coefficients were interpreted following the suggestions of Lorenzo and ten Berge (2006) that values between .85 and .94 indicate a fair similarity and those higher than .95 a good factor similarity.

Finally, an attempt was made to replicate the DSM-5 personality trait model [7, 16] using Goldberg’s approach, with which successive models with an increasing number of factors can be estimated [25]. This method examines the hierarchical structure of a set of variables based on factor scores from rotated solutions. In accordance with Goldberg’s recommendations, we conducted a one-factor extraction followed by a series of varimax rotated principal analyses with two to five factors. Regression-based factor scores were estimated for each solution. In particular, at the top level is the first unrotated principal factor (FUPC), the second level consists of a two-factor solution, the third of a three-factor one, and so on until the five-factor solution is reached which is under consideration in the current study. In this study, factor loadings of .30 or higher were used for the interpretation of the factors at each level. The correlations between orthogonal factor scores were viewed as path coefficients in a hierarchical structure. In particular, we examined these correlations at each level with those of the level below to construct a hierarchical representation.

## Results

### Unidimensionality of the 25 facets and the five domains

Parallel analyses supported a one-factor structure for each of the facet scales apart from Depressivity, Callousness and Perceptual Dysregulation. For the Depressivity scale, the first three eigenvalues from the actual data were 6.91, 1.20, and 0.80; the corresponding first three 95^th^ percentile random data eigenvalues were 1.14, 1.11, and 1.09, suggesting the retention of two components for rotation. Exploratory factor analysis of Depressivity items revealed that the fourteen items scored strongly on the first component (mean factor loading .67, range .49 to .79) and minimally on the second (mean factor loading .16, range .00 to .47). For the Callousness scale, the first three eigenvalues from the actual data were 5.02, 1.15, and 0.95; the corresponding first three 95^th^ percentile random data eigenvalues were 1.14, 1.11, and 1.08, suggesting the retention of two components. Exploratory factor analysis of Callousness items revealed that the fourteen items scored strongly on the first component (mean factor loading .53, range .28 to .77) and minimally on the second (mean factor loading .14, range .01 to .31). For the Perceptual Dysregulation scale, the first three eigenvalues from the actual data were 4.09, 1.15, and 0.91; the corresponding first three 95^th^ percentile random data eigenvalues were 1.13, 1.10, and 1.07, suggesting the retention of two components. Exploratory factor analysis of Perceptual Dysregulation items revealed that the twelve items scored strongly on the first component (mean factor loading .52, range .36 to .68) and minimally on the second (mean factor loading .16, range .01 to .49).

Parallel analyses also supported a one-factor structure for each of the five domain scales apart from Negative Affectivity. The first three eigenvalues from the actual data were 3.03, 1.22, and 0.97; the corresponding first three 95^th^ percentile random data eigenvalues were 1.09, 1.06, and 1.03, suggesting the retention of two components for rotation to solution. Exploratory factor analysis of the seven Negative Affectivity facet scales revealed that six of the seven facets loaded strongly on the first component (mean factor loading .62, range .37 to .77) and minimally on the second (mean factor loading .13, range .02 to .27). In particular, it appeared that Restricted Affectivity was the only facet to score on the second factor.

Cronbach’s α computed for the 25 facets and the five domains from the initial construction of the personality inventory are displayed in Table 2. For the 25 facets, internal consistency indices were good, with indices ranging from .68 at the lowest level for Suspiciousness and Irresponsibility to .95 for Eccentricity, with a mean internal consistency of .82 for the facet scales. For the five domains, they were also good, with indices ranging from .75 for Negative Affectivity to .82 for Antagonism and Disinhibition, with a mean internal consistency of .80.

**Table 2:** Item-level reliability indices (α), means (M) and standard deviations (sd) for the 25 facets and the 5 domains.

		Roskam et al.		Krueger et al. (2012)		
	*α*	*M*	*sd*	*M*	*sd*	*d*
Anhedonia	.81	.77	.55	.89	.64	.20
Anxiousness	.87	1.39	.72	1.02	.73	-.51
Attention seeking	.87	.87	.63	.81	.65	-.09
Callousness	.85	.49	.41	.40	.50	-.19
Deceitfulness	.83	.99	.49	.52	.54	-.91
Depressivity	.92	.66	.57	.53	.62	-.21
Distractibility	.88	1.03	.65	.82	.69	-.31
Eccentricity	.95	.79	.69	.82	.76	.04
Emotional lability	.85	1.31	.70	.94	.74	-.51
Grandiosity	.76	.56	.53	.82	.58	.46
Hostility	.82	1.11	.57	.91	.67	-.32
Impulsivity	.85	.96	.65	.77	.57	-.31
Intimacy avoidance	.81	.54	.58	.61	.65	.11
Irresponsibility	.68	.62	.46	.39	.49	-.48
Manipulativeness	.83	.80	.67	.80	.67	.00
Perceptual dysregulation	.81	.62	.48	.44	.48	-.37
Perseveration	.77	.99	.52	.82	.62	-.29
Restricted affectivity	.77	.97	.59	.97	.56	.00
Rigid perfectionism	.88	1.07	.64	1.05	.68	-.03
Risk taking	.89	1.24	.55	1.05	.66	-.31
Separation insecurity	.78	1.13	.71	.80	.68	-.47
Submissiveness	.78	.91	.63	1.17	.66	.40
Suspiciousness	.68	.70	.50	.95	.58	.46
Unusual beliefs	.80	.57	.55	.64	.63	.12
Withdrawal	.86	.73	.54	1.01	.72	.44
Negative affectivity	.75	1.11	.40	1.07	.44	-.09
Detachment	.81	.74	.41	.78	.54	.08
Antagonism	.82	.68	.41	.61	.46	-.16
Disinhibition	.82	.98	.35	1.06	.30	.24
Psychoticism	.80	.67	.49	.64	.57	-.05

Means and standard deviations of the 25 facet scales and the five domains are presented in Table 2. To facilitate comparison, the mean and standard deviation of the PID-5 facet scales from the representative sample described in Krueger et al. (2012) are displayed. Deceitfulness was especially low in the current sample in comparison with Krueger et al. (2012), with *d* of-.91. Variations at a medium effect size (.30 to .50) were also displayed for eleven scales. Small effect sizes were displayed for the five domain scales. Intercorrelations between the five domain means displayed in Table 3 ranged from .29 between Detachment and Antagonism to .64 between Negative Affectivity and Detachment.

**Table 3:** Rotated factor loadings for five-factor solution.

	I	II	III	IV	V
Anxiousness	**.79**	.14	-.04	.06	.09
Emotional Lability	**.67**	-.05	-.01	.28	.24
Hostility	**.36**	.21	**.39**	.29	.12
Perseveration	**.63**	.29	**.30**	.14	.30
Restricted Affectivity	-.07	.**63**	.29	.00	.09
Separation Insecurity	**.67**	-.12	.12	.11	.00
Submissivenes	**.42**	.13	.12	-.04	-.03
Anhedonia	**.46**	**.65**	-.01	.14	.01
Depressivity	**.63**	**.48**	-.03	.25	.12
Intimacy Avoidance	.05	**.54**	.07	.06	.12
Suspiciousness	**.46**	**.35**	.16	.13	.20
Withdrawal	.22	**.76**	.07	-.04	.12
Attention Seeking	.27	-.17	**.56**	.15	.13
Callousness	.01	**.42**	**.59**	.24	.08
Deceitfulness	.15	.19	**.76**	.23	-.00
Grandiosity	.02	.16	**.61**	-.06	.25
Manipulativeness	.05	.11	**.79**	.09	.11
Distractibility	**.36**	.26	.07	**.52**	.20
Impulsivity	.17	-.04	.25	**.58**	.18
Irresponsibility	.16	.25	**.36**	**.52**	.07
Rigid Perfectionism	**.46**	.16	.20	**-.36**	.23
Risk Taking	-.21	-.07	**.31**	**.33**	.26
Eccentricity	.24	**.34**	.21	**.34**	**.51**
Perceptual Dysregulation	**.40**	.25	.16	**.33**	**.58**
Unusual Beliefs and Experiences	.16	.18	.25	.10	**.64**
Factor intercorrelations					
Negative Affectivity	1.00	.64	.40	.59	.55
Detachment		1.00	.29	.44	.54
Antagonism			1.00	.53	.47
Disinhibition				1.00	.60
Psychoticism					1.00

*Note*. Factor loadings >|.30| are in bold. I = Negative Affectivity, II = Detachment, III = Antagonism, IV = Disinhibition, V = Psychoticism

### Factor structure replication

Parallel analyses conducted on the 25 facet scales supported a five-factor structure. The first seven eigenvalues from the actual data were 8.29, 2.97, 2.45, 1.50, 1.16, 1.09 and 0.93; the corresponding first seven 95^th^ percentile random data eigenvalues were 1.20, 1.17, 1.15, 1.13, 1.11, 1.10 and 1.08, suggesting the retention of five components for rotation to solution.

An exploratory factor analysis followed by equamax oblique rotation of the 25 PID-5 facet scales showed reasonable fit indices of χ² = 3567.27, *df* = 185, *p* < .000, CFI of .89, GFI of .99 and RMR of .03. The loading parameter estimates for the standardized five-factor solution are presented in Table 3. All the facets but two had their primary loading on the expected factor. Depressivity loaded on firstly Negative Affectivity and secondly on Detachment. Restricted Affectivity was associated with Detachment rather than with Negative Affectivity. As previously observed, a number of scales (Hostility, Perseveration, Anhedonia, Depressivity, Suspiciousness, Callousness, Distractibility, Irresponsibility, Rigid Perfectionism, Risk Taking, Eccentricity and Perceptual Dysregulation) significantly cross-load.

Factor congruency coefficients comparing the current five-factor structure and the initial one [6] were as follows: Negative Affect, .86; Detachment, .91; Antagonism, .95; Disinhibition, .93; and Psychoticism, .97. They suggest good to high factor structure similarity for all domains.

### DSM-5 personality trait model replication

**Fig 1:**
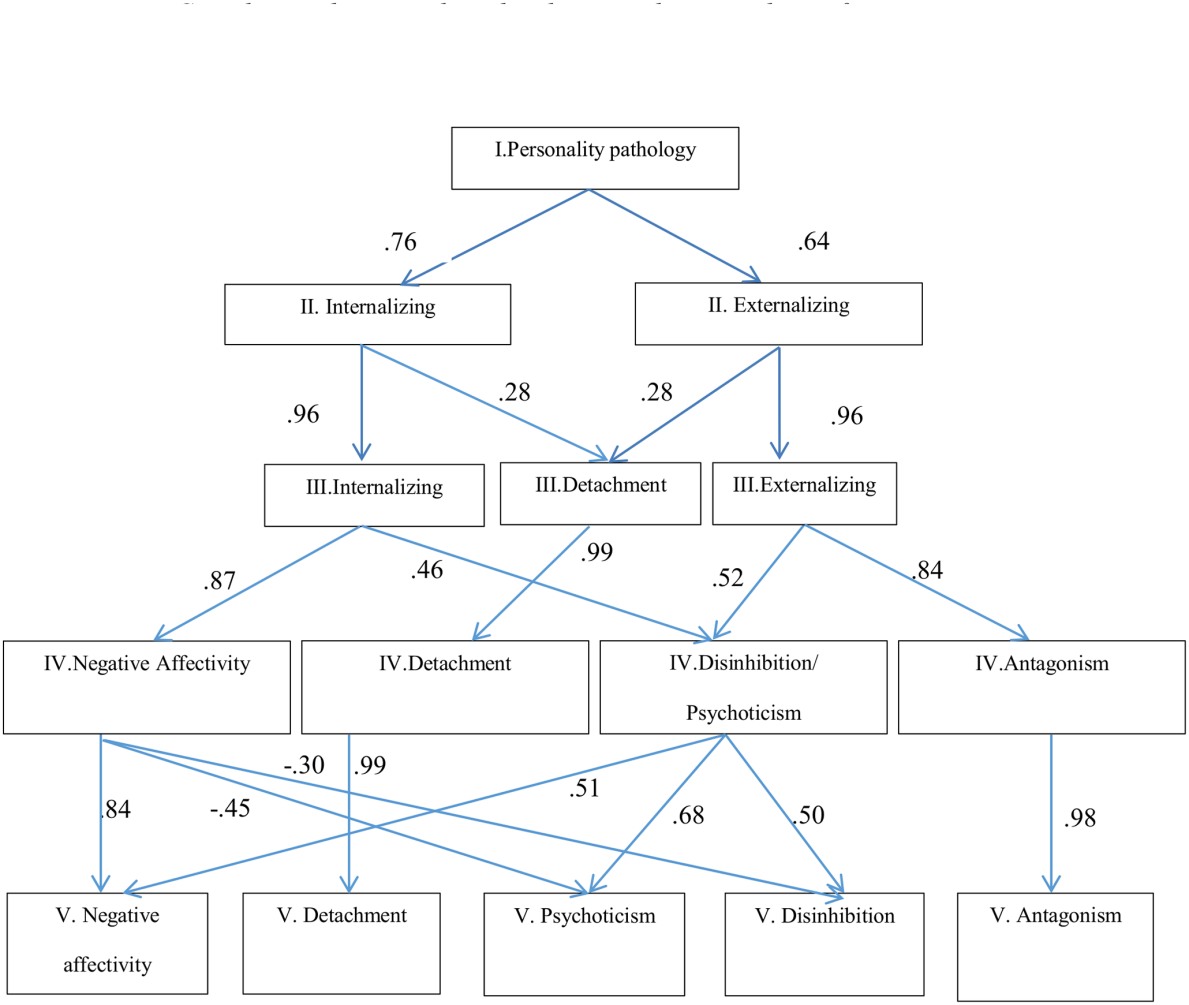
Correlations between the subordinate and superordinate factors. Fig 1. displays the hierarchical structure of the French version of the PID-5.

The hierarchical structure of the French version of the PID-5 is presented in Fig 1. Paths with values >.20 are reported. In the one-factor solution, each of the 25 facets loaded at >.40 except for Submissiveness (.35) and Risk taking (.20). As in a previous study [15, 16], these results suggest that the FUPC captures overall “personality pathology”. Two factors emerged from the FUPC.

Thirteen facets loaded (>.30) and had their primary loading on the first factor, labelled Internalizing, encompassing the Negative Affectivity (except for Hostility) and Detachment domains plus Distractibility, (lack of) Rigid Perfectionism and Perceptual Dysregulation. The twelve other facets from the Antagonism, Disinhibition and Psychoticism domains loaded (>.30) and had their primary loading on the second factor, labelled Externalizing.

Moving to the three-factor solution, Internalizing and Externalizing factors mainly maintained their structure, but the facets from the Detachment domain, i.e. Withdrawal, Intimacy Avoidance and Anhedonia emerged as a separate factor. Restricted Affectivity, which has been seen to be associated with Detachment rather than with Negative Affectivity in the current study, also had meaningful loading on this third factor.

In the four-factor solution, the Internalizing factor split into Negative Affectivity and a second factor composed of the Disinhibition and Psychoticism domains. The Externalizing factor also split into Antagonism and the Disinhibition/Psychoticism factor, whereas Detachment maintained its structure.

Finally, as displayed in the EFA, the five-factor solution replicated the factorial structure from the initial version of PID-5 [6]. The Disinhibition/Psychoticism factor split into two factors, the first labelled Disinhibition and characterized by significant loadings of Rigid Perfectionism, Irresponsibility, Distractibility, and Impulsivity, and the second labelled Psychoticism and characterized by meaningful loadings of Unexpected Beliefs and Experiences, Eccentricity, Perceptual Dysregulation, but also Risk Taking. The hierarchical structure of DSM-5 traits and path coefficients between higher- and lower-order factors is shown in Fig 1. The loadings of facets on factors for the Bass-Ackwards analysis are presented in the S1 Table.

## Discussion

The main objective of the current study was to present data about the factorial structure of the French version of the Personality Inventory for DSM-5 (PID-5). In particular an attempt was made to test the assumption of unidimensionality of the 25 facets and the five domains, to replicate both the factorial structure of the English version of the PID-5 [5] and the DSM-5 trait model [7, 16]. In a large sample of French-speaking European participants, we provided evidence for the unidimensionality of many of the 25 facets and the five domains by means of parallel analyses and reliability indices. However, unidimensionaility was questionable for three facets, Depressivity, Callousness and Perceptual Dysregulation and one domain, i.e. Negative Affectivity. Even when two components were suggested for retention, EFA indicated that items strongly loaded on the first factor and minimally on the second. Internal consistency indices were also good. In this respect, the current results produced similar conclusions to those of previous studies reviewed. Moreover, in line with Quilty et al. (2013), descriptive statistics from the current sample were compared with those reported by Krueger et al. (2012) in the initial validation study. Except for Deceitfulness, which was lower in the French-speaking sample, variations at only low to medium effect sizes were displayed. These slight variations suggest that the French version of the PID-5 produces scores in the same range as those found with the English version.

With regard to the five-factor structure replication, in line with the study of Fossati et al. (2013), parallel analyses suggested that five factors had to be retained. And goodness-of-fit indices from the EFA confirmed that a five-factor structure was acceptable. As in previous EFA research, we found cross-loadings and primary or meaningful loadings (>.30) were on the expected factors for 24 of the 25 facets. In particular, Restricted Affectivity loaded on Detachment as in Wright et al. (2012), Zimmerman et al. (2014), and Bo et al. (2015). As expected, congruence coefficients indicated that we had replicated the factorial structure displayed by Krueger et al. (2012). Current congruence coefficients from .86 to .97 were hence in the same range as those reported in previous studies, with the initial factorial structure of Krueger et al. (2012) ranging from .82 to .98 [9, 13–15, 17].

With regard to the hierarchical structure of the PID-5 studied with Goldberg’s procedure, we found a two-level structure corresponding to Internalizing and Externalizing as in the three previous relevant studies [8, 15, 16]. This two-factor solution matches a model of general psychopathology [22]. At the three-factor level, Detachment came from both Externalizing and Internalizing factors rather than from Internalizing only, and Negative Affect did not clearly appear as was the case in Bo et al. (2015), Morey et al. (2013) and Wright et al. (2012). At the fourth level, our results were very similar to those displayed by Bo et al. (2015) and Wright et al. (2012), with Negative Affectivity, Detachment, Disinhibition/Psychoticism and Antagonism resembling the four factors of the DAPP-BQ, labelled Emotional Dysregulation, Inhibitedness, Dissocial Behavior, and Compulsivity [28, 29]. Finally, going through successive models with an increasing number of factors, we replicated the five expected factors, as had previously been done [15, 16].

In sum, in the context of an optimal translation process which has been validated by the American authors themselves, of a large European sample, of statistical analyses which were similar to those used in previous PID-5 validation studies, the current study points to a replication of the unidimensionality of the 25 facets and the five domains as well as to a replication of the five-factor structure as initially proposed in the PID-5. Our intention was to make the validated French version of the PID-5 self-report form available for both clinicians and researchers. It contributes effectively to the research on the dimensional conceptualization of pathological personality. However, the current study is by no mean definitive. A first limitation is that it is limited to data on the factor structure of the French version of PID-5. Future studies should report on convergent validity, discriminant properties and test-retest reliability. Another limitation relates to the sample composition, which consisted predominantly of female students. Studies conducted with the French items are needed with clinically referred participants. Finally, in order to ensure comparability with previous studies, we used statistical procedures that have been commonly employed to study facet and domain unidimensionality, five-factor structure replication and hierarchical structure. Although this may be considered appropriate in a first attempt to validate the French version of the PID-5, future attempts should be made, for example, to measure equivalence across nationalities and recruitment sites, using a more sophisticated statistical approach.

## Supporting Information

S1 File Dataset. (XLS)

S1 Table Loadings of facets on factors for the Bass-Ackwards analysis. (DOCX)
