# Supplementary material for: The Psychometric Properties of the French Version of the Personality Inventory for DSM-5
Source: PLoS One. 2015 Jul 20;10(7):e0133413. doi: 10.1371/journal.pone.0133413 (PMC4508106; doi:10.1371/journal.pone.0133413)
Supplement: S1 Table — (DOCX) [file pone.0133413.s002.docx]

S1 Table. Loadings of facets on factors for the Bass-Ackwards analysis

|  | One-factor solution | Two-factor solution | | Three-factor solution | | | Four-factor solution | | | | Five-factor solution | | | | |
| --- | --- | --- | --- | --- | --- | --- | --- | --- | --- | --- | --- | --- | --- | --- | --- |
|  | FUPC | Int | Ext | Int | DET | Ext | NA | DET | DIS/PSY | ANT | NA | DET | PSY | DIS | ANT |
| Anxiousness | .57 | .81 | -.06 | .82 | .06 | -.06 | .77 | .13 | .26 | -.09 | .80 | .10 | -.18 | -.08 | -.03 |
| Emotional Lability | .56 | .70 | .05 | .78 | -.16 | .13 | .63 | -.08 | .51 | -.06 | .81 | -.10 | .05 | .10 | .01 |
| Hostility | .66 | .43 | .51 | .43 | .16 | .50 | .29 | .18 | .45 | .37 | .47 | .19 | .21 | .08 | .40 |
| Perseveration | .76 | .75 | .29 | .73 | .24 | .25 | .62 | .28 | .39 | .18 | .73 | .27 | -.03 | .00 | .23 |
| Restricted Affectivity | .43 | .15 | .48 | -.04 | .72 | .29 | -.08 | .70 | .03 | .34 | -.07 | .71 | .05 | -.00 | .31 |
| Separation Insecurity | .44 | .57 | .01 | .67 | -.24 | .10 | .69 | -.20 | .23 | .09 | .66 | -.21 | -.29 | .04 | .17 |
| Submissivenes | .35 | .44 | .02 | .43 | .10 | .01 | .55 | .09 | -.07 | .16 | .33 | .09 | -.62 | .06 | .23 |
| Anhedonia | .63 | .70 | .15 | .55 | .59 | .00 | .43 | .65 | .24 | -.04 | .46 | .64 | -.22 | .13 | .00 |
| Depressivity | .71 | .82 | .14 | .74 | .38 | .06 | .57 | .46 | .42 | -.07 | .69 | .45 | -.14 | .17 | -.00 |
| Intimacy Avoidance | .40 | .30 | .27 | .12 | .65 | .09 | .00 | .67 | .13 | .07 | .08 | .67 | .05 | .05 | .05 |
| Suspiciousness | .65 | .62 | .27 | .56 | .33 | .21 | .45 | .37 | .32 | .15 | .57 | .36 | .08 | -.03 | .16 |
| Withdrawal | .55 | .51 | .25 | .30 | .78 | .03 | .24 | .80 | .07 | .08 | .25 | .79 | -.07 | -.07 | .06 |
| Attention Seeking | .44 | .14 | .52 | .24 | -.22 | .62 | .28 | -.26 | .24 | .62 | .28 | -.23 | -.06 | .09 | .68 |
| Callousness | .59 | .14 | .75 | .05 | .44 | .65 | -.05 | .41 | .30 | .60 | .07 | .44 | .17 | .15 | .60 |
| Deceitfulness | .60 | .14 | .77 | .12 | .19 | .75 | .11 | .15 | .26 | .75 | .12 | .19. | -.06 | 21 | .80 |
| Grandiosity | .44 | .02 | .65 | -.01 | .23 | .61 | .07 | .16 | .03 | .73 | .05 | .18 | .14 | -.20 | .70 |
| Manipulativeness | .51 | .01 | .78 | .01 | .15 | .77 | .04 | .08 | .17 | .82 | .05 | .12 | .07 | .03 | .83 |
| Distractibility | .63 | .56 | .33 | .55 | .15 | .32 | .24 | .23 | .68 | .01 | .52 | .25 | .06 | .53 | .10 |
| Impulsivity | .48 | .23 | .46 | .32 | -.17 | .55 | .02 | -.11 | .73 | .19 | .38 | -.08 | .37 | .48 | .26 |
| Irresponsibility | .61 | .30 | .59 | .29 | .19 | .57 | .05 | .21 | .58 | .33 | .24 | .25 | -.00 | .60 | .42 |
| Rigid Perfectionism | .41 | .46 | .09 | .41 | .21 | .04 | .60 | .19 | -.19 | .28 | .46 | .16 | -.03 | -.64 | .23 |
| Risk Taking | .21 | -.19 | .54 | -.13 | -.11 | .59 | -.34 | -.11 | .47 | .34 | -.04 | -.07 | .57 | .26 | .34 |
| Eccentricity | .71 | .49 | .52 | .45 | .31 | .46 | .19 | .36 | .60 | .23 | .49 | .37 | .35 | .19 | .26 |
| Perceptual Dysregulation | .75 | .62 | .44 | .60 | .21 | .42 | .36 | .27 | .62 | .18 | .64 | .27 | .30 | .16 | .22 |
| Unusual Beliefs and Experiences | .57 | .34 | .48 | .31 | .21 | .45 | .17 | .23 | .41 | .33 | .40 | .23 | .46 | -.09 | .31 |

*Note* : FUPC = first unrotated principal factor ; Int = Internalizing ; Ext = Extrernalizing ; DET = Detachment ; NA = Negative Affectivity ; DIS = Disinhibition ; PSY= Psychoticism ; ANT = Antagonism.
